# Glycan Microarray-Assisted Identification of IgG Subclass Targets in Schistosomiasis

**DOI:** 10.3389/fimmu.2018.02331

**Published:** 2018-10-09

**Authors:** Y. Y. Michelle Yang, Angela van Diepen, Katarzyna Brzezicka, Niels-Christian Reichardt, Cornelis H. Hokke

**Affiliations:** ^1^Department of Parasitology, Leiden University Medical Center, Leiden, Netherlands; ^2^Glycotechnology Laboratory, Centro de Investigación Cooperativa en Biomateriales (CIC biomaGUNE), San Sebastián, Spain; ^3^Centro de Investigación Biomédica en Red en Bioingeniería, Biomateriales y Nanomedicina (CIBER-BBN), San Sebastián, Spain

**Keywords:** glycan antigens, schistosomiasis, IgG subclass, glycan-microarray, fucosylation

## Abstract

Infection with schistosomes is accompanied by the induction of antibodies against the parasite. Despite having IgG against both protein and glycan antigens, infected individuals remain chronically infected until treated, and re-infection is common in endemic areas as immunity does not develop effectively. Parasite specific IgG subclasses may differ in functionality and effectivity with respect to effector functions that contribute to parasite killing and immunity. In this study, we investigated if specific IgG subclasses target specific antigenic schistosome glycan motifs during human infection. Sera from 41 *S. mansoni* infected individuals from an endemic area in Uganda were incubated on two glycan microarrays, one consisting of a large repertoire of schistosome glycoprotein- and glycolipid- derived glycans and the other consisting of chemically synthesized core xylosylated and fucosylated N-glycans also expressed by schistosomes. Our results show that highly antigenic glycan motifs, such as multi-fucosylated terminal GalNAc(β1-4)GlcNAc (LDN) can be recognized by all IgG subclasses of infection sera, however with highly variable intensities. Detailed examination of core-modified N-glycan targets revealed individual antibody responses specific for core-xylosylated and core α3-fucosylated glycan motifs that are life stage specifically expressed by schistosomes. IgG1 and IgG3 were detected against a range of N-glycan core structures, but IgG2 and IgG4, when present, were specific for the core α3-fucose and xylose motifs that were previously found to be IgE targets in schistosomiasis, and in allergies. This study is the first to address IgG subclass responses to defined helminth glycans.

## Introduction

Schistosomiasis is a parasitic infection of humans and mammals contracted by exposure to schistosome cercariae shed by infected aquatic snails. Once the parasite establishes itself in a human host, the infection which is associated with debilitating pathology due to tissue-deposition of eggs remains chronic until treated. In the schistosomiasis endemic areas in Africa, South-America, and South-East Asia, reinfection after treatment is common. Longitudinal studies have shown that resistance to re-infection with schistosomes develops very slowly. Many years of exposure to schistosomes and multiple treatments are required for the immune response to become effective (1–3). Various studies have shown that anti-schistosome antibodies are pivotal for anti-parasite immunity. Passive immunization with serum or monoclonal antibodies against schistosome antigens (4–7) were shown to reduce worm burden and egg production in previously unexposed mice upon challenge with schistosomes. Moreover, transfer of sera and of purified IgG from animals immunized with the protective Sm-p80 antigen conferred resistance to challenge infection, and it has been shown that the level of protection induced by immunization with Sm-p80 is reduced in antibody deficient mice (8).

As schistosomes are highly glycosylated organisms that express many glycan motifs different from mammals, it is not surprising that an abundance of antibodies are generated against schistosomal protein- and lipid-linked glycans exposed to the host (9–14). It is becoming increasingly clear that glycan antigens play an important role in helminth infection immunology, but it remains ambiguous whether anti-glycan antibodies contribute positively or negatively to protection.

Previously we have studied the anti-glycan IgG and IgM responses in schistosome infected rhesus macaques that are able to expel the worms (15), in order to identify glycan targets of antibodies that might be involved in the self-cure mechanism. We showed that serum IgG antibodies against highly fucosylated schistosome glycoproteins and glycolipids formed during infection were sustained when the macaques cleared the infection, indicating that these antibodies are present in a protective context and possibly play a role in the killing of adult worms in the host. Additionally, sera from macaques containing high titres of IgG against highly fucosylated motifs were able to kill schistosomula *in vitro* (15) and it has been shown that a monoclonal antibody specific for the fucosylated LeX antigen was protective in a mouse model of *S. mansoni* infection (6). On the other hand, humans that are generally susceptible to reinfection also have high titres of IgG antibodies against highly fucosylated motifs (16), indicating that anti-glycan antibodies are not necessarily protective. Indeed, some studies have regarded schistosome glycans to form a smokescreen that diverts the host immune system from attacking vulnerable *peptide* epitopes that could mediate protective responses (17–19). It has however been appreciated that protective *glycan* epitopes may also occur, but these could be under-represented and masked by irrelevant or “smokescreen” epitopes (20). As a consequence, protective anti-schistosome glycan responses may be difficult to identify. These observations called for investigations into differential recognition of specific schistosome glycan motifs in different models and cohorts such as reported in several recent glycan microarray-assisted studies (12–16, 21).

Alternatively, research into the IgG subclass-specific response toward defined glycan antigens may shed light on the role of anti-glycan antibodies in infection. Perhaps the key to associate glycan antigens with immunity is not in the antigen itself, but rather by the type of antibody response generated. Particular IgG subclasses have been found to correlate with resistance or susceptibility to schistosome infection: IgG1 against schistosome surface antigens were observed in putatively resistant individuals in a Brazilian cohort (22), while individuals that were chronically infected lacked high IgG1 against the same antigens, but mounted high IgG4 toward various schistosome antigens instead. IgG4, as well as IgG2 have been found to correlate with susceptibility of schistosome reinfection (23, 24). On the other hand, human serum IgG1 and IgG3, as well as IgE have been found to be potent inducers of eosinophil-mediated cytotoxicity, while IgG4 antibodies were found to be inhibiting cytotoxicity (25) and compete with IgE to prevent antigen cross-linking and IgE mediated effector function (26). So far, studies on IgG subclass response against schistosome antigens have focused on schistosome surface proteins (22) or complex ill-defined schistosome antigen mixtures such as SEA or AWA (27). Little is known however about IgG subclass reactivity to defined schistosome glycan antigens. Interestingly, a recent study has shown that anti-glycan IgE responses are highly restricted toward only a few specific epitopes out of the many glycan antigens expressed by schistosomes, in particular N-glycan core-xylose or core α3-fucose, motifs that are often associated with plant glycans and allergens (28).

In the current study, we address the question whether specific serum IgG subclass responses to defined antigenic glycans occur during human schistosome infection, and whether these might be restricted to certain motifs or not. To this end, we tested the IgG subclass responses in a selection of 41 serum samples from a relatively homogeneous population in terms of exposure and infection intensity (16, 29) toward a large collection of schistosome glycan antigens. Using two glycan microarrays, one comprising a large set of native glycans isolated from different schistosome life-stages (14–16), the other comprising a set of synthetic N-glycans with different core and branch modifications previously described (21) we determined for each of the IgG subclasses which glycan motifs are targeted during infection, and evaluated whether these are different or not for IgG1-4. Our results indicate that in schistosomiasis sera, each of the IgG subclasses can be directed against a variety of antigenic glycan motifs, but with different patterns for the different individuals tested. To our knowledge, this is the first study that addresses IgG subclass responses to defined glycans and glycan motifs in a helminth infection.

## Materials and methods

### Sera

Sera used in this study were described in an earlier publication (16) where 41 schistosome infected individuals were selected from an original cohort study in a highly prevalent *S. mansoni* endemic area (29). The cohort study obtained ethical approval from the Uganda National Council for Science and Technology (UNCST) and was supported by the Cambridge Local Research Ethics Committee. Selected subjects had an age range of 5 to 46 years old, all with patent *S. mansoni* infection. The geometric mean of the intensity of infection measured by egg per gram feces was 560.65 (CI_95%_: 343.88, 914.05).

### Glycan microarray construction and incubation

A glycan microarray constructed of glycans derived from *S. mansoni* cercariae (75 N-glycan fractions and 102 O-glycan fractions), adult worms (77 N-glycan fractions and 31 O-glycan fractions) and eggs (57 egg N-glycan fractions and 98 soluble egg antigen O-glycan fractions), and 30 glycans derived from glycosphingolipids representing multiple life stage, was described previously (14, 16). Twenty-four blank spots with spot buffer were included for array background control. Each glycan fraction was immobilized on a glass slide in triplicate. The synthetic glycan microarray containing a collection of core-xylosylated and core-α3 and -α6 fucosylated N-glycans with various core extensions has been described previously (21).

Binding assays of individual or pooled sera on both arrays were conducted following the protocol as described previously (14–16). Briefly, the microarray slide was blocked with 2% BSA, 50 mM ethanolamine in PBS. Serum samples were diluted 1:100 in PBS-0.01% Tween20 with 1% BSA. All four mouse anti-human isotype antibodies were purchased from Invitrogen. IgG1 and IgG3 were labeled with the Promokine PF-647 labeling kit; IgG2 and IgG4 were labeled with the PF-555 labeling kit following the manufacturer's protocol. All anti-human IgG subclass antibodies were diluted 1:200 in PBS-0.01 Tween20 to detect bound serum antibodies on the slide. All washing steps were performed with successive rinses with PBS-0.05% Tween20 and with PBS. The last washing step was finished by an additional wash with milliQ water and the slides were dried and kept in the dark until scanning.

### Scanning and data analysis

A G2565BA scanner (Agilent Technologies, Santa Clara, CA) was used to scan the slides for fluorescence at 10 μm resolution using lasers at 532 and 633 nm. Anti-IgG2 and anti-IgG4 antibodies were detected at 532 nm and anti-IgG1 and anti-IgG3 antibodies at 633 nm. Data and image analysis was performed with GenePix Pro 7.0 software (Molecular Devices, Sunnyvale, CA). Spots were aligned and re-sized using round features with no CPI threshold. Background-subtracted median intensities were averaged per time point and processed as described by Oyelaran et al. (30). Datasets were log_2_ transformed to remove the basic trends of variance. A hierarchical clustering analysis (HCA, complete linkage clustering using Euclidean distance metric) was performed to group associated glycan fractions using MultiExperiment Viewer v4.5.

## Results

### Serum IgG subclass response against glycans expressed by schistosomes in infected individuals

**Figure 1:**
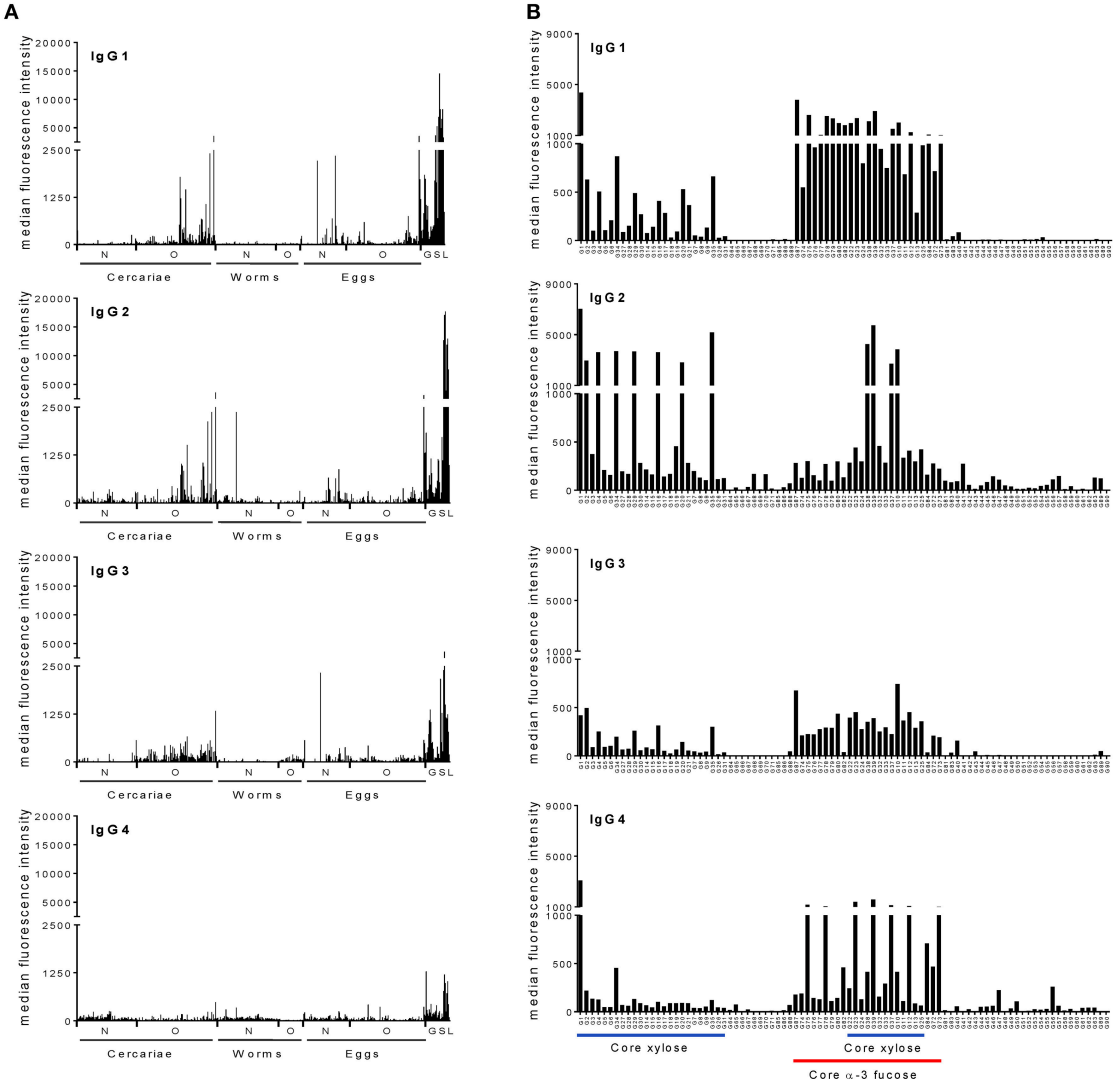
Schistosome infected individuals produce IgG subclass antibodies to schistosome glycans. Averaged serum IgG subclass response from schistosome-infected individuals to **(A)** complex N-, O-, and glycoshingolipid glycans isolated from different life stages of schistosomes and **(B)** core modified N-glycans synthesized chemically. The horizontal axis indicates different glycan structures. Each bar corresponds to antibody binding to individual glycan fractions printed on the glycan microarray. Schistosome GSL glycans are shown as a group irrespective of the life stage. N, N-glycans; O, O-glycans; GSL, Glycoshingolipid derived glycans.

To investigate whether IgG subclasses in sera from a schistosome-infected cohort react with specific motifs or subsets of parasite-associated glycans, we determined IgG1, IgG2, IgG3. and IgG4 binding intensities against a large variety of glycans in pooled sera of 41 infected individuals using glycan microarrays. These sera were from a relatively homogeneous population in terms of exposure and infection intensity). All individuals had patent *S. mansoni* infection (geometric mean infection intensity (epg) was 560.65 (CI_95%_: 343.88, 914.05). We found that in the pool IgG of all four subclasses are present against a wide range of schistosome-derived N-, O-, and glycosphingolipid glycans printed on the array (Figure 1A). IgG1 and IgG2 bound to the various glycans with similar high intensities, while anti-glycan IgG4 binding intensities were lowest out of the four subclasses. Glycan targets for each of the IgG subclasses consisted mainly of highly fucosylated LDN epitopes, abundantly expressed on glycolipid-derived glycans and cercarial O-glycans (31). Interestingly, IgG subclass binding of N-glycan core xylose (abundant on glycans from cercariae and miracidia) and core α3-fucose epitopes (abundant on glycans from eggs and miracidia), as determined by use of the synthetic glycan array, were rather different across the four subclasses (Figure 1B). IgG1, IgG3, and IgG4 in pooled infection serum showed higher binding intensities to core α3-fucose containing structures than to structures containing only core-xylose, with IgG4 being restricted to different subsets of the α3-fucose containing structures. In contrast, IgG2 response in this pool was mainly directed toward core xylose containing N-glycans. Core α3-fucose has been shown to be an antigenic target in schistosome-infected humans, mice and rhesus macaques (12, 15). Interestingly, alone or in combination with core xylose, core α3-fucose also forms the major cross-reactive carbohydrate epitopes that are IgE targets on a variety of plant-derived allergens (28, 32, 33). To further analyse the differential reaction of specific IgG subclasses with core-modified N-glycans, we investigated the IgG subclass response in each infected individual on the synthetic glycan array.

### Schistosome infected individuals differentially express IgG subclass antibodies toward schistosome glycans

Among the individual sera incubated on the synthetic glycan array a striking variability in IgG subclass response toward the core-modified glycans was observed (Figure S1). Most sera contained antibodies binding to core α3-fucose and core xylose modified N-glycans. However, this was not a universal response and no clear correlation between IgG subclass and glycan motifs was observed.

### IgG subclass response profile of schistosome infected individuals

**Figure 2:**
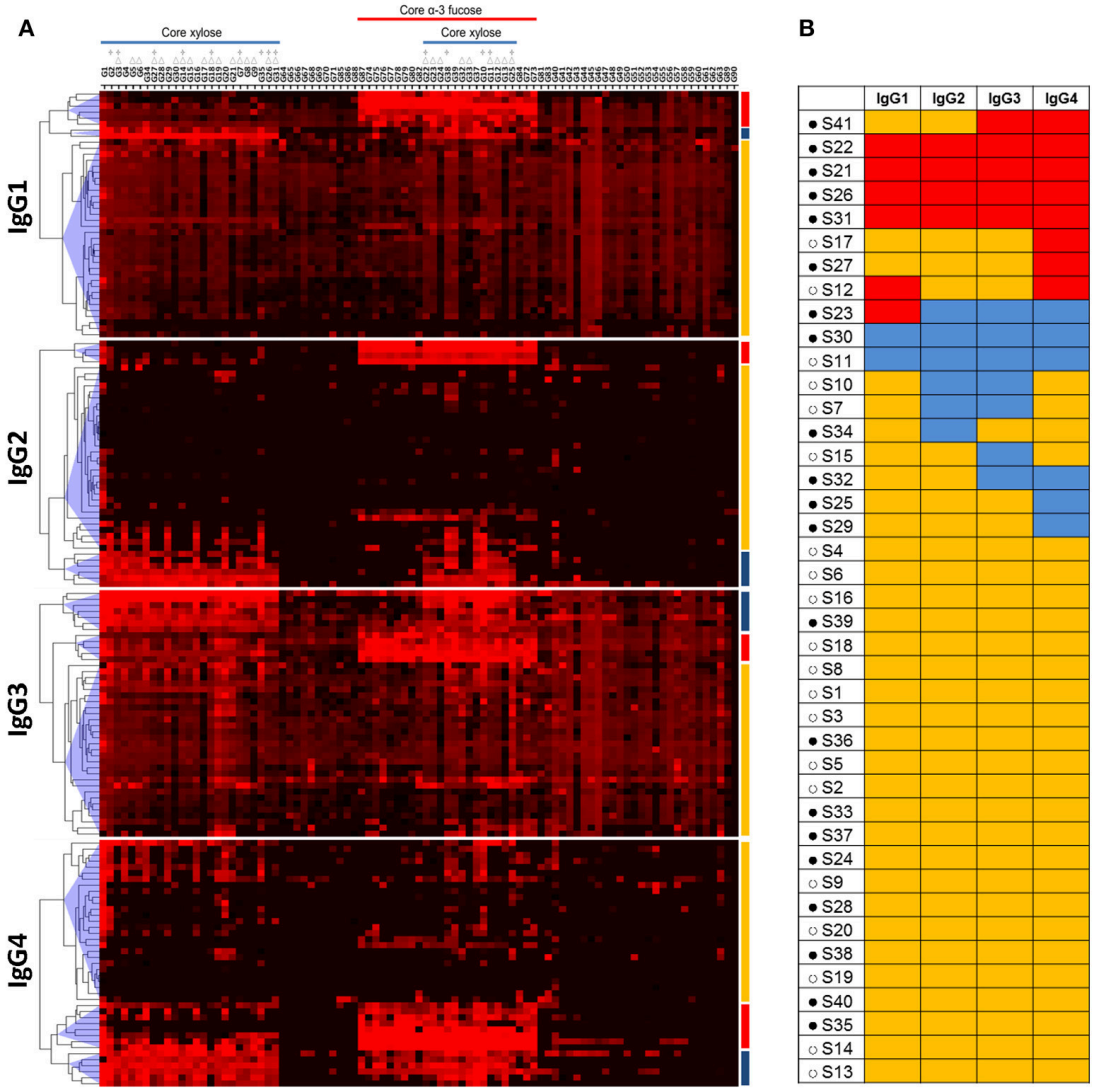
Hierarchical clustering analysis of anti-glycan antibody responses in schistosome infected individuals. **(A)** Heatmap showing IgG subclass response of schistosome infected subjects (columns) to core modified N-glycan fractions (rows) that have been synthesized and described by Brzezicka et al. Median fluorescence intensity was corrected for baseline and log_2_ transformed; increase in antibody binding is indicated by the red color intensity. Three major clusters of subjects were identified for each IgG subclass based on antibody binding intensity to glycans: one cluster of individuals had high IgG binding to core α3-fucose (red), another cluster of individuals to core xylose (blue), and one cluster of individuals without specific binding to core α3-fucose or core xylose (yellow). Core xylosylated and core α-3 fucosylated structures are indicated. Within core-xylose containing structures, those that have additional monosaccharides on the α-3mannose (Δ) and those that miss the core α-6mannose (*) are indicated. **(B)** IgG subclass response profile of each schistosome infected individual. Red: high response against core α3-fucose; blue: high response against core xylose; yellow: low response against other glycans. Filled circles (•): age 12 and under. Open circles (°): age 20 and older.

In view of the variations in IgG subclass responses to the core-modified N-glycans between individuals, we performed a hierarchical clustering analysis and grouped the individuals based on response patterns for each IgG subclass (Figure 2A). We observed a cluster of individuals that had specific and high IgG1, 2, 3, or 4 binding to core α3-fucose (cluster red). Another cluster of individuals had high IgG binding to core xylose containing structures (cluster blue), while the third cluster, with the highest number of individuals, only had lower amounts of IgG binding to either core α3-fucose or core xylose (cluster yellow). When comparing the IgG subclass distribution for each motif within each serum, we saw that some individuals had high binding to core α3-fucose with all four IgG subclasses (cluster red in all subclasses) (Figure 2B), while other individuals responded strongly to core α3-fucose structures with only one or two subclasses that were variably of the IgG1, 2, 3, or 4 type. Similarly, two individuals of the 41 individuals responded strongly to core xylose containing N-glycans with all IgG subclasses (cluster blue in all subclasses), while other individuals responded only with one or two subclasses, variably of the IgG2, IgG3, or IgG4 class. These observations indicate that IgG subclass responses toward these glycan antigens are not restricted by the nature of the antigen, but rather appear to be related to each individual response to the infection. One interesting observation was that most of the individuals that had high IgG subclass response toward either core xylose or core α3-fucose were children aged 12 or younger (Figure 2B).

### Structural motifs bound by IgG1−4

**Figure 3:**
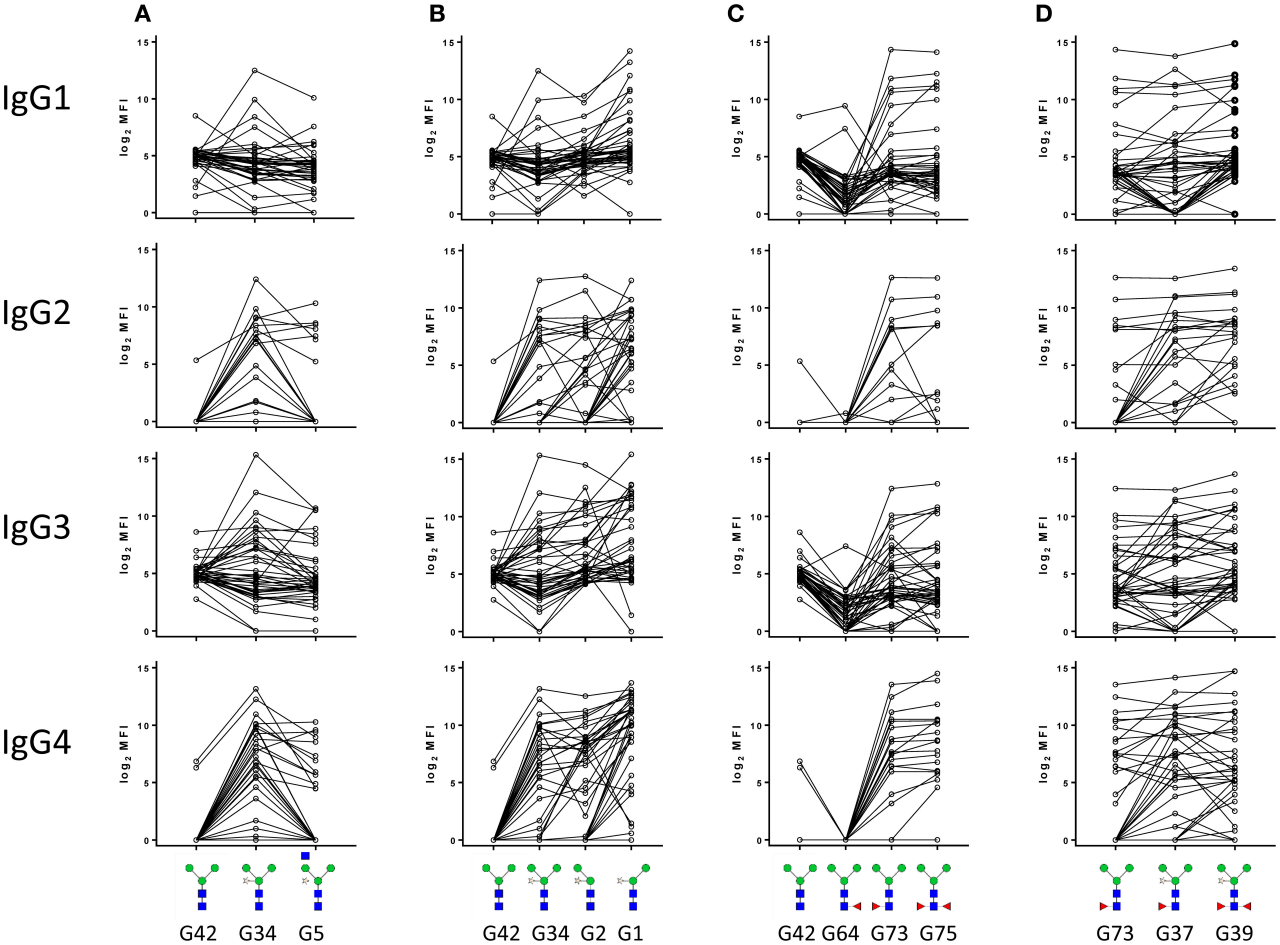
IgG subclass response of schistosome infected individuals to core xylose **(A,B)** and core α3 fucose **(C,D)** in the presence or absence of adjacent structural elements. Median fluorescence intensity was corrected for baseline and log_2_ transformed. The code for each structure as described in the original publication (21) is indicated.

Next, to gain more insight in the structural determinants that are recognized specifically by the different IgG subclasses, we examined in detail how the binding of IgG1, IgG2, IgG3, and IgG4 to core α3-fucose and core xylose is influenced by adjacent structural elements. Comparison of the signal intensities of four sets of structurally related synthetic glycan structures first revealed that moderate levels of IgG1 and IgG3 reactive with the unsubstituted trimannosyl core glycan (Man_3_GlcNAc_2_) (G42) were detected in most sera tested (Figures 3A–C). In contrast, IgG2 and IgG4 to the N-glycan core were only detected when either a xylose (G34) or an α3-fucose (G73) residue were present (Figures 3A,C). Interestingly, IgG1 and IgG3 in general did not become more reactive upon addition of core xylose to the trimannosyl core, but signal intensities did increase significantly upon α3-fucosylation while core α6-fucose addition (G64) had the opposite effect of lowering the IgG1 and IgG3 response to the otherwise unsubstituted trimannosyl core. The addition of α6-fucose had no effect on the reactivity of any of the four IgG subclasses to the α3-fucosylated core however (G64) (Figure 3C). Regarding the combination of both the antigenic core xylose and core α3-fucose modifications it appears that sera where IgG2 and IgG4 reactivity is negative against core α3-fucose becomes positive upon addition of the xylose (G37) (Figure 3D). Since reactivity is also observed to core-xylosylated glycans without fucose, it appears that α3-fucose and xylose form independent antigenic motifs. Finally, IgG2 and IgG4, but not IgG1 and IgG3 reactivity against core xylose are hindered by the addition of a GlcNAc branch to the 3-linked Man (G5), but removal of the 3-linked Man (G1) enhances reactivity, indicating that reactivity to xylose depends on its spatial accessibility (Figures 3A,B). Together these data suggested that IgG2 and IgG4 reactivity to N-glycan core structures in schistosomiasis sera are either against the core xylose and core α3-fucose motifs, whereas IgG1 and IgG3 are less specific and restricted.

## Discussion

In this study we have investigated the IgG subclass responses against defined parasite-derived glycans in schistosome-infected individuals. Detailed glycan microarray analysis indicated that IgG1, 2, 3, and 4 in schistosome infection sera are variably present against a wide range of antigenic parasite glycans. Interestingly, individuals infected with schistosomes respond with particularly high variability to the antigenic α3-fucose and β2-xylose core modifications of N-glycans with respect to the different IgG subclasses. This variability in antibody response was not a reflection of the intensity of infection as all sera included this study were from heavily infected individuals living in a schistosome endemic area. The sera used in this study were selected from a larger study (29), where selected individuals had an age range between 5 to 46 years old, all with patent *S. mansoni* infection, with fecal egg counts between 343.88 and 914.05 epg (CI_95%_ of geometric mean). Previously, we have used this set of sera to compare the anti-glycan (total) IgG and IgM in schistosome infected children and adults (16) and found that although there were anti-glycan antibody differences between children and adults, other factors apart from age also played a role in shaping anti-glycan antibody response profiles. It is well-known that the overall serum IgG subclass distribution is generally different between adults and children. IgG1 and IgG3 development is faster and reaches around 75% of the adult serum levels at the age of five, while IgG2 and IgG4 levels rise much slower, reaching 70% of adult serum levels at the age of 14. Nevertheless, large inter-individual variations were observed previously for IgG subclass development (34), as corroborated by our study. Although most individuals with responses of any subclass toward core modified glycans were children, many children did *not* produce antibodies against core modified N-glycans, raising the question which other factors, possibly cross-reactive antigens, determine the induction of these schistosome-reactive antibodies.

The sample selection used here to study the glycan specificity of IgG subclasses in schistosomiasis infection serum is not suitable for addressing immunoepidemiological questions. Nonetheless, the variable subclass responses to specific glycan antigens observed in this study may have the potential to reflect the immune profile of infected individuals and act as markers for infection with respect to intensity, exposure, chronicity, immunopathology or resistance to infection. High IgG1 titers against a specific set of schistosome tegument protein antigens for instance have been observed in putatively resistant individuals in a Brazilian cohort but not in chronically infected individuals, where high IgG4 antibodies against the same schistosome antigens were observed instead (22). In addition, IgG1 and IgG3 against protective schistosome antigens has also been found in naturally resistant individuals but not in chronically infected or unexposed individuals (35). These studies indicate the importance of IgG1 against protective antigens in developing immunity. In contrast, susceptibility to reinfection in humans has been associated with high levels of IgM, IgG2, and IgG4 (23, 24, 26, 27). The presence of IgM, IgG2, and IgG4, purified from serum of infected individuals, prevented eosinophil mediated killing of schistosomula by other IgG subclasses present in human infection sera (25). The IgG4 subclass is usually produced after repeated, long-term antigenic stimulation, and has minimal effector functions. Literature describes highly elevated IgG4 toward schistosome antigens (26) that may compete with IgE and prevent antigen cross-linking and IgE mediated effector functions. Interestingly, although we have not observed very high IgG4 binding intensities against schistosome glycans, precisely the core α3-fucose and core xylose motifs that form the cross-reactive carbohydrate determinants for IgE in plant allergens as well as helminths (28, 33, 36) are also the only IgG4 reactive glycan elements in the tested N-glycans in the current study. It should be noted that expression of core α3-fucose and core xylose motifs during the schistosome life cycles appears to be highly specific. By mass spectrometric glycomics approaches it has been shown that core xylose is abundant on N-glycans of cercariae and miracidia (31), whereas core α3-fucose has been detected in abundance in N-glycans on the secretory egg glycoproteins IPSE/α1 (37) and omega-1 (together with core α6-fucose) (38) and on N-glycans derived from the miracidia (31) and the egg glycoprotein kappa-5 (together with α6-fucose and core xylose) (39). Both core xylose and core α3-fucose are expressed by plant glycoproteins (33, 40) and in a small subset of other helminths (36). The specific IgG determined in our study may therefore also have been triggered by other antigen sources other than schistosomes. It would be interesting to investigate the IgG subclass response of infected individuals toward more complex glycan antigens that are schistosome specific, such as highly fucosylated LDN epitopes on glycoproteins and glycosphingolipids of the parasite and its secretions. Such complex schistosome antigens, induce higher total IgG binding in schistosome infected rhesus macaques compared to core modified epitopes (15). Given that highly fucosylated motif such as Fucα1-2Fucα1-3 on LDN have not been found in organisms other than schistosomes, it would be interesting to see if different individuals would have a less variable IgG subclass distribution to these complex, more parasite-specific glycan antigens, when these would become available as synthetic, defined antigens.

In this study we have for the first time investigated the IgG subclass response against schistosome glycans in infected individuals. The complex interplay between how antigens trigger IgG subclass response calls for analysis with a larger human cohort with better defined resistant and susceptible immune profiles, to be able to understand whether particular subclasses against particular glycan epitopes are associated with a disease state. Although high inter-individual antibody variation was observed against core modified N-glycans expressed by schistosomes, IgG subclass response against complex antennae schistosome glycans remain to be elucidated.

## Author contributions

YY and KB performed experiments. YY, KB, and AvD analyzed data. AvD, N-CR, and CH designed and supervised the study. YY, AvD, and CH wrote the paper.

### Conflict of interest statement

The authors declare that the research was conducted in the absence of any commercial or financial relationships that could be construed as a potential conflict of interest.
