# Supplementary material for: Glycan Microarray-Assisted Identification of IgG Subclass Targets in Schistosomiasis
Source: Front Immunol. 2018 Oct 9;9:2331. doi: 10.3389/fimmu.2018.02331 (PMC6190862; doi:10.3389/fimmu.2018.02331)
Supplement: Supplementary file 1 [file Image_1.pdf]

Fig. S1

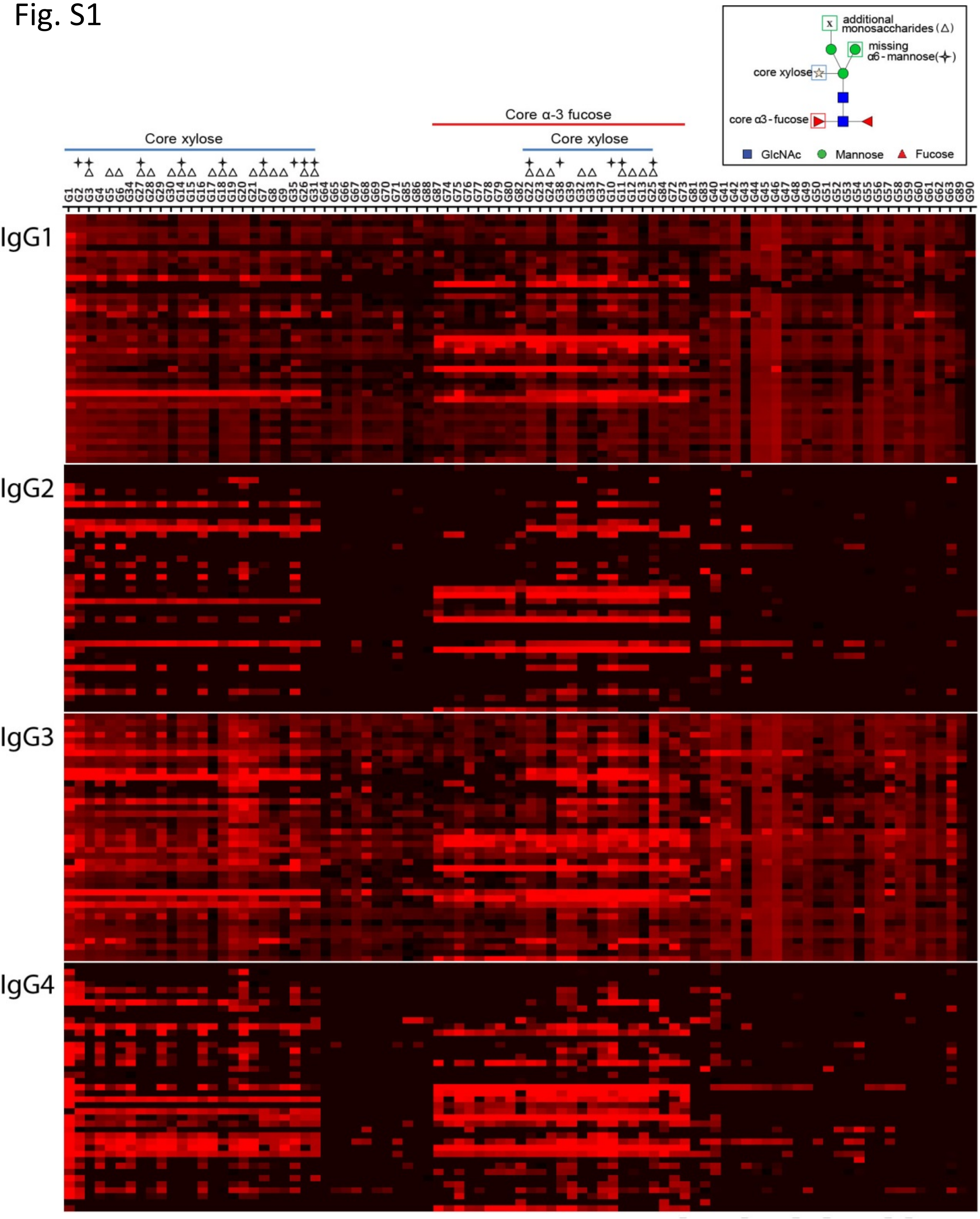

Supplementary figure S1. Hierarchical clustering analysis of anti-glycan IgG1, IgG2, IgG3 and IgG4 responses in *S. mansoni* infected individuals. Heatmap showing IgG subclass response of schistosome infected subjects to core modified N-glycans that have been synthesized and described by Brzezicka *et al.*. Median fluorescence intensity was corrected for baseline and log_2 transformed; increase in antibody binding is indicated by the red color intensity. The glycan code for each structure (Brzezicka et al., 2015) is indicated and core xylosylated and core α-3 fucosylated structures are marked. Within core-xylose containing structures, those that have additional monosaccharides on the α-3 mannose (Δ) and those that miss the core α-6 mannose (*) are indicated. The order in which the individual anti-glycan responses are shown is kept the same for all four subclasses.
